# Supplementary material for: Expanding the microbiologist toolbox via new far-red-emitting dyes suitable for bacterial imaging
Source: Microbiol Spectr. 2023 Dec 14;12(1):e03690-23. doi: 10.1128/spectrum.03690-23 (PMC10782969; doi:10.1128/spectrum.03690-23)
Supplement: Supplemental Data — Figures S1 to S14; Tables S1 and S2; captions of Videos S1 and S2; general information for analytical data; analytical data for KK 1518, KK 1116, and KK 1905-NHS; supplemental references. [file spectrum.03690-23-s0001.pdf]

Supplementary Data
for

Expanding the microbiologist toolbox *via* new far-red emitting dyes suitable for bacterial imaging

Massimiliano Lucidi[a,b], Giulia Capecchi[a], Daniela Visaggio[a,b,c], Tecla Gasperi[a], Miranda Parisi[d], Gabriella Cincotti[d], Giordano Rampioni[a,c], Paolo Visca[a,b,c], Kirill Kolmakov[e]

[a] Department of Science, University Roma Tre, viale G. Marconi 446, 00146 Rome, Italy

[b] NBFC, National Biodiversity Future Center, piazza Marina 61, 90133 Palermo, Italy

[c] IRCCS Fondazione Santa Lucia, via Ardeatina 306/354, 00179 Rome, Italy

[d] Department of Engineering, University Roma Tre, via V. Volterra 62, 00146 Rome, Italy

[e] glyXera GmbH, Brenneckestraße 20 ZENIT II/Haus 66, D-39120 Magdeburg, Germany

Correspondence to:

Dr. Massimiliano Lucidi, Department of Science, University Roma Tre, Viale G. Marconi 446, 00146 Rome, Italy massimiliano.lucidi@uniroma3.it;

Dr. Kirill Kolmakov, glyXera GmbH, Brenneckestraße 20 ZENIT II/Haus 66, D-39120 Magdeburg, Germany kirill_kolmakov@mail.ru; k.kolmakov@glyxera.com.

This PDF file includes:

- Supplementary Figures S1 to S14
- Tables S1 and S2
- Captions of Supplementary Videos S1 and S2
- General information for analytical data
- Analytical data for KK 1518, KK 1116, and KK 1905-NHS
- Supplementary references

Other Supplementary Data for this manuscript include:

- Supplementary Videos S1 and S2

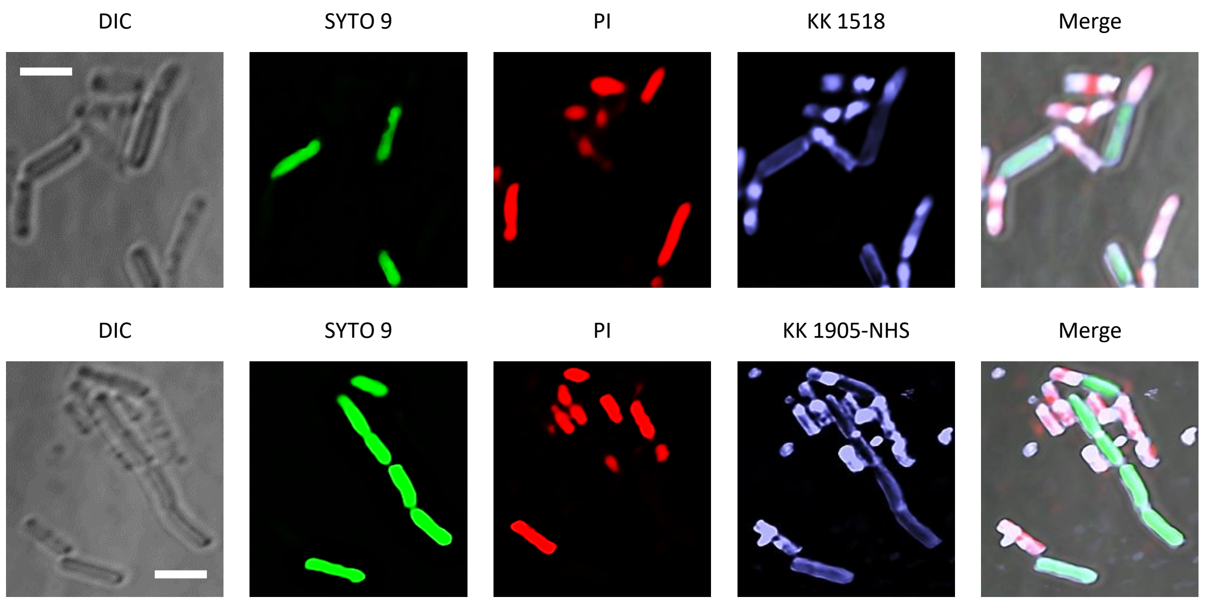

Fig. S1. Representative CLSM images of *B. subtilis* cells harvested from the death phase (48 h of incubation at 37°C in LB) stained with the indicated dyes. Fluorescence, DIC, and fluorescence-DIC merged channels are shown. Scale bar: 2.5 μm.

	Fluorescence fold-induction		
Fluorophore	Phospholipid staining	Protein staining	Nucleic acid staining
**KK 114S**	< 20.0%	110.4%	< 20.0%
**KK 114-NHS**	24.2%	80.0%	< 20.0%
**KK 1115**	25.3%	72.6%	< 20.0%
**KK 1116**	25.0%	90.3%	< 20.0%
**KK 1517**	1293.2%	140.1%	53.4%
**KK 1517-NHS**	< 20.0%	114.4%	< 20.0%
**KK 1518**	1212.2%	34.3%	208.9%
**KK 1558-NHS**	< 20.0%	41.5%	< 20.0%
**KK 1905**	< 20.0%	< 20.0%	121.7%
**KK 1905-NHS**	1828.8%	< 20.0%	123.6%
**STAR RED**	< 20.0%	26.1%	< 20.0%
**PI**	34.1%	< 20.0%	4164.7%
**NR**	2362.2%	1161.1%	83.5%

Fig. S2. POPC-based liposomes, BSA, and DNA staining using the fluorophores employed in this work. 5 mg/mL of POPC-based liposomes, 1 mg/mL of BSA, and 100 ng/µL of *E. coli* MG1655 genomic DNA were stained with the indicated fluorophores at a final concentration of 10 µM. Then, the emitted fluorescence was measured using a Sparks 10M multilabel plate reader (Tecan) selecting the appropriate excitation and emission wavelengths for each molecule. The autofluorescence of liposomes, BSA, and DNA at the selected excitation and emission wavelengths was subtracted from each recorded emitted fluorescence. Fluorescence fold-induction (FFI) was calculated by dividing the dye's fluorescence in the presence of POPC-based liposomes, BSA, or DNA, and the dye's fluorescence in the absence of the indicated macromolecules. Values of FFI above 20% were considered predictive of fluorescence emission increase in the presence of liposomes, BSA, or DNA. Data are the average of three independent experiments.

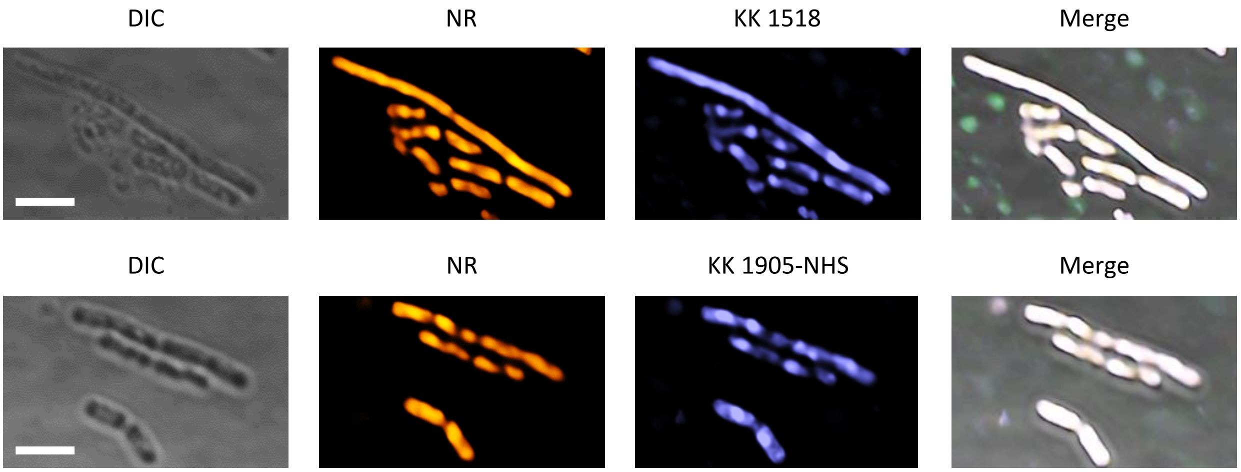

Fig. S3. Representative CLSM images of *B. subtilis* cells heat-killed and stained with the indicated dyes. Fluorescence, DIC, and fluorescence-DIC merged channels are shown. Scale bar: 2.5 μm.

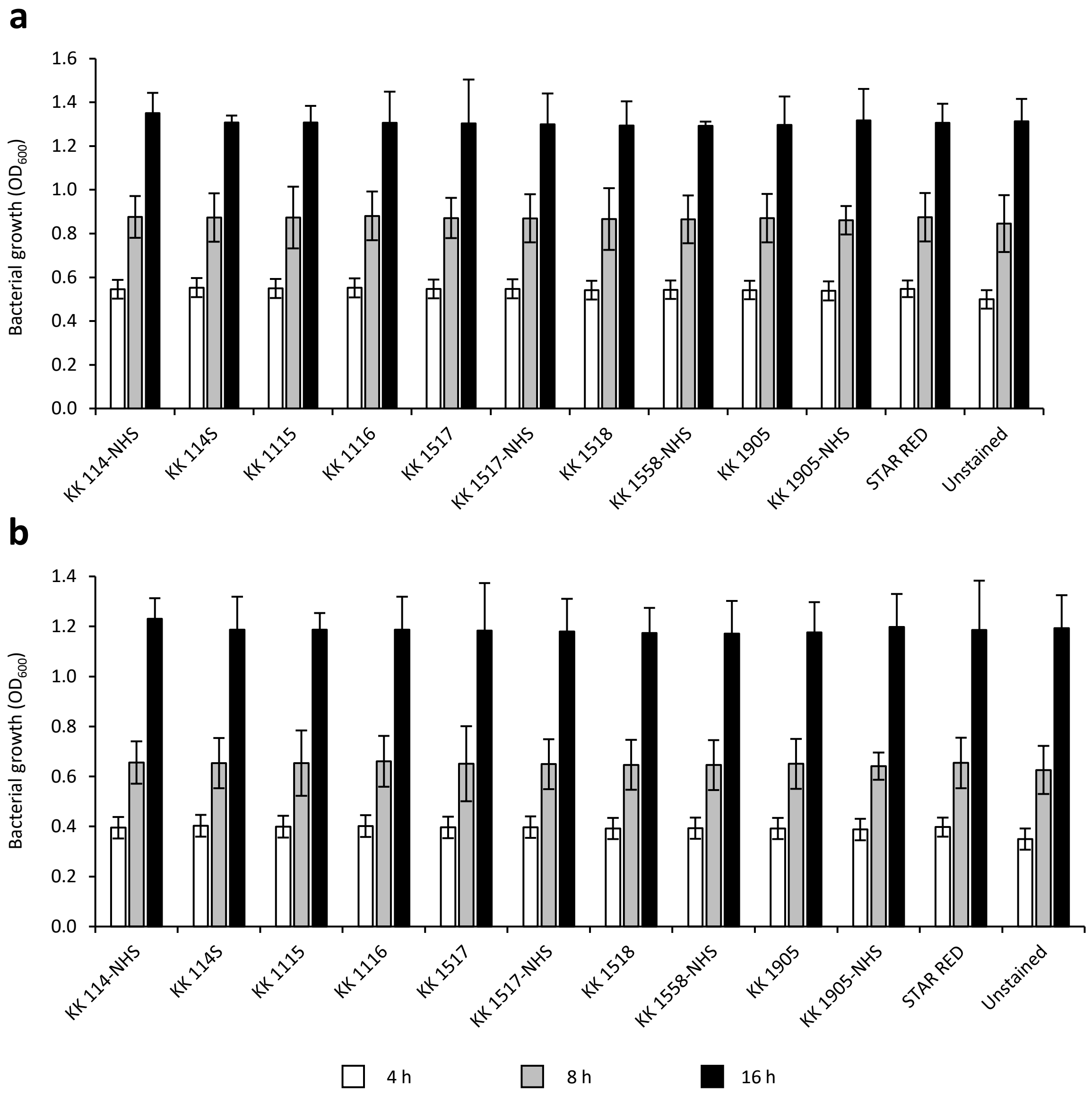

Fig. S4. Growth levels of *B. subtilis* (**a**) and *E. coli* (**b**) labeled with the dyes used in this work. *B. subtilis* and *E. coli* cells were inoculated in LB and incubated for 18 h at 37 °C. Then, bacterial cells were suspended in LB with or without 10 µM of the indicated dye, and the OD_{600} was monitored during time using a Sparks 10M multilabel plate reader (Tecan) for up to 16 h at 37 °C. Three representative time points of the bacterial growth curves are shown. Data are the means ± SD of three independent experiments.

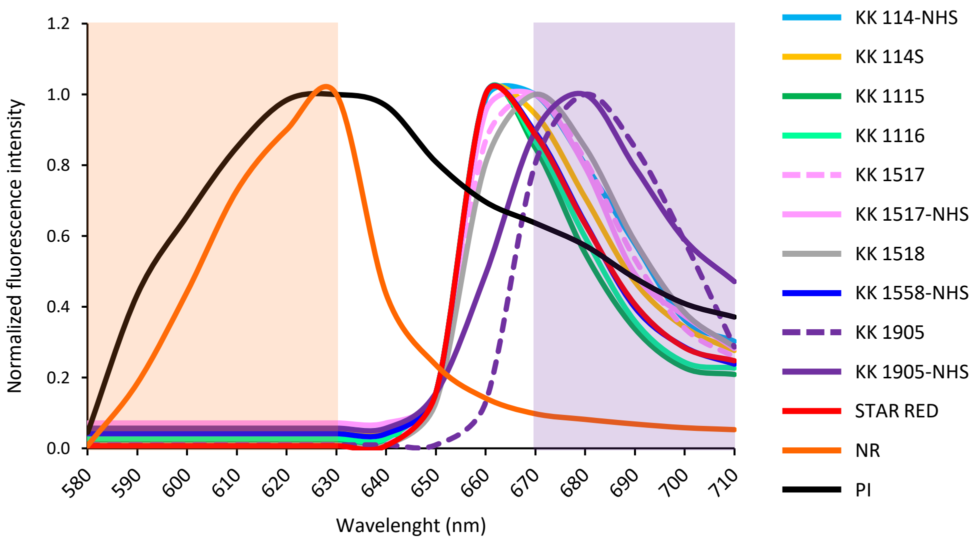

Fig. S5. Emission spectra of bacterial cells labeled with the dyes used in this work. Whole-cell emission spectra were determined on heat-inactivated *E. coli* cells after staining with the indicated dye, by the Nikon A1+ CLSM lambda scan equipped with 100× oil immersion objective (NA 1.4). All spectra were normalized at a maximum value of 1.0 to facilitate the comparison and are representative of three spectra giving similar profiles. Orange and violet boxes indicate the emission bandwidths employed to minimize bleed-through artifacts of NR and KK 1905-NHS, respectively.

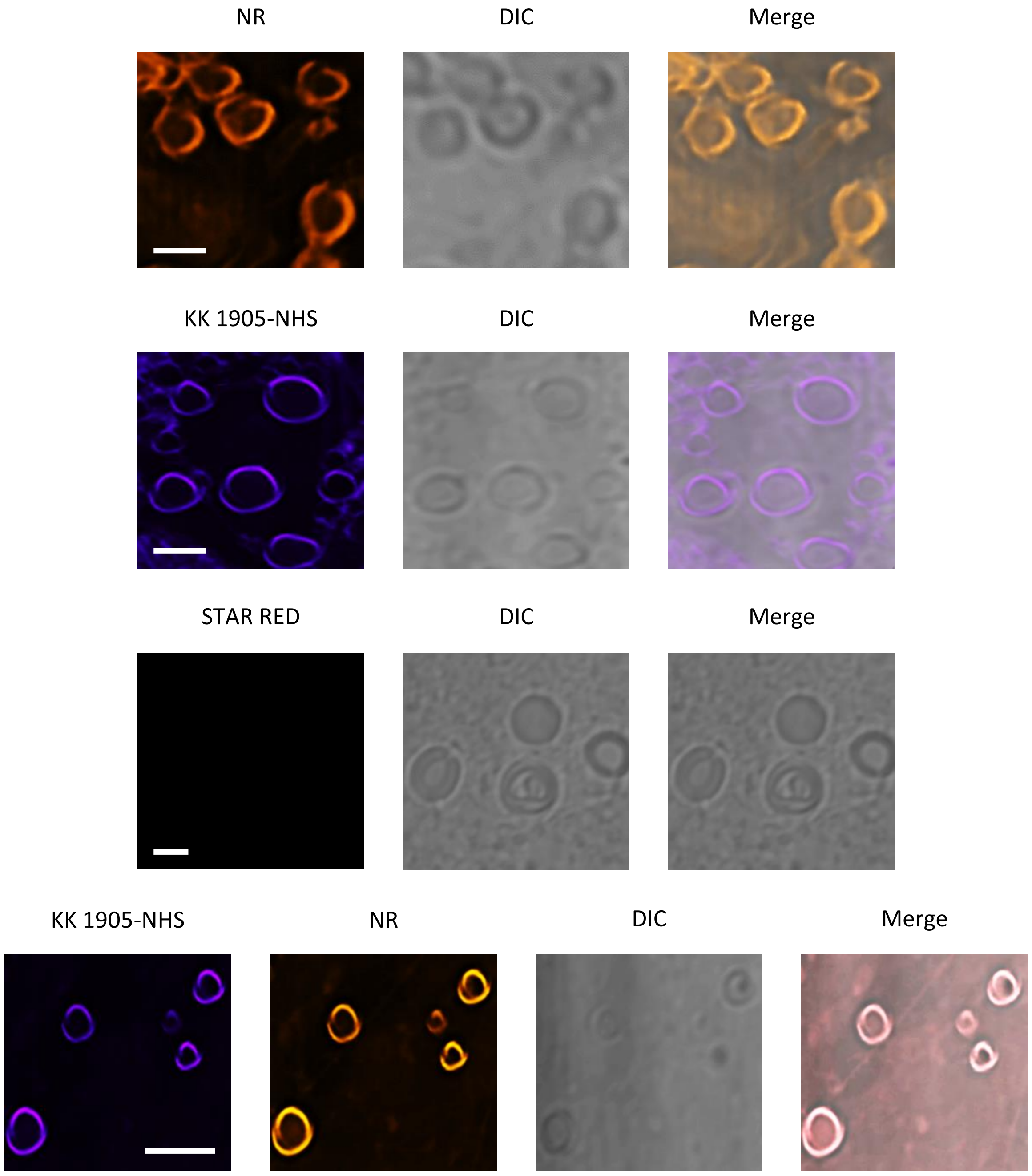

Fig. S6. Representative CLSM images of POPC-based liposomes stained with the indicated dyes. Fluorescence, DIC, and fluorescence-DIC merged channels are shown for each dye. Scale bar: 2.5 μm.

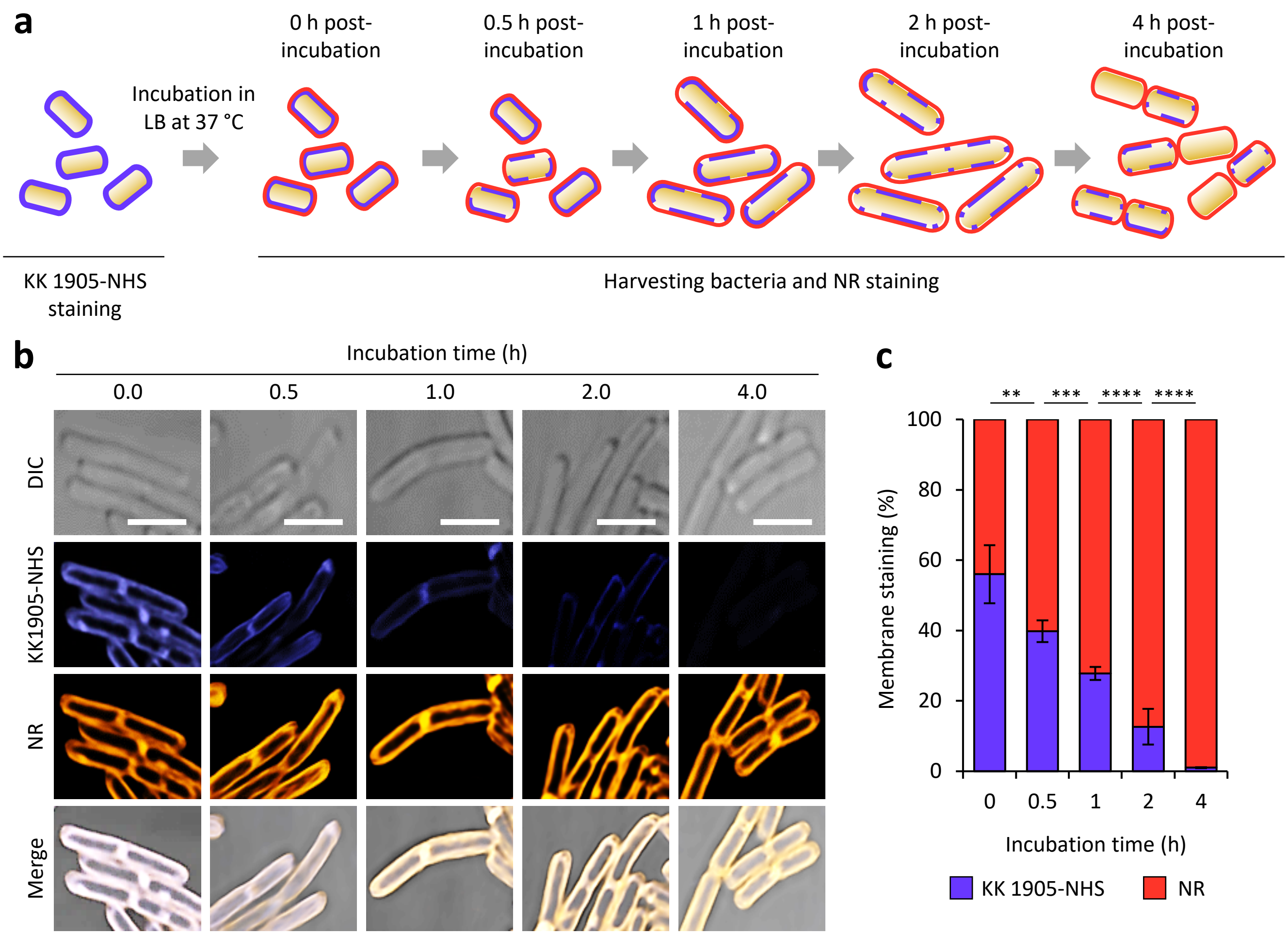

Fig. S7. Monitoring the bacterial membrane biogenesis with reverse two-step labeling. (**a**) Schematic representation of the reverse two-step labeling procedure. *B. subtilis* cells were stained with KK 1905-NHS and incubated at 37 °C in LB. At 0, 0.5, 1, 2, and 4 h-post incubation, bacteria were harvested, stained with NR, and visually inspected using CLSM. Violet and orange cell outlines indicate the KK 1905-NHS-labeled "old" and the NR-labeled membranes, respectively. (**b**) Representative images of bacterial membranes obtained using the Nikon A1+ CLSM equipped with a 100× oil immersion objective. Scale bar: 2.5 µm. (**c**) For the indicated time points, NR and KK 1905-NHS-associated fluorescence was quantified for different bacterial cells (n = 50). Asterisks indicate statistically significant differences in NR and KK 1905-NHS fluorescence percentages in the analyzed cell membranes between the indicated time points (** P < 0.01; *** P <0.001 **** P <0.0001).

a

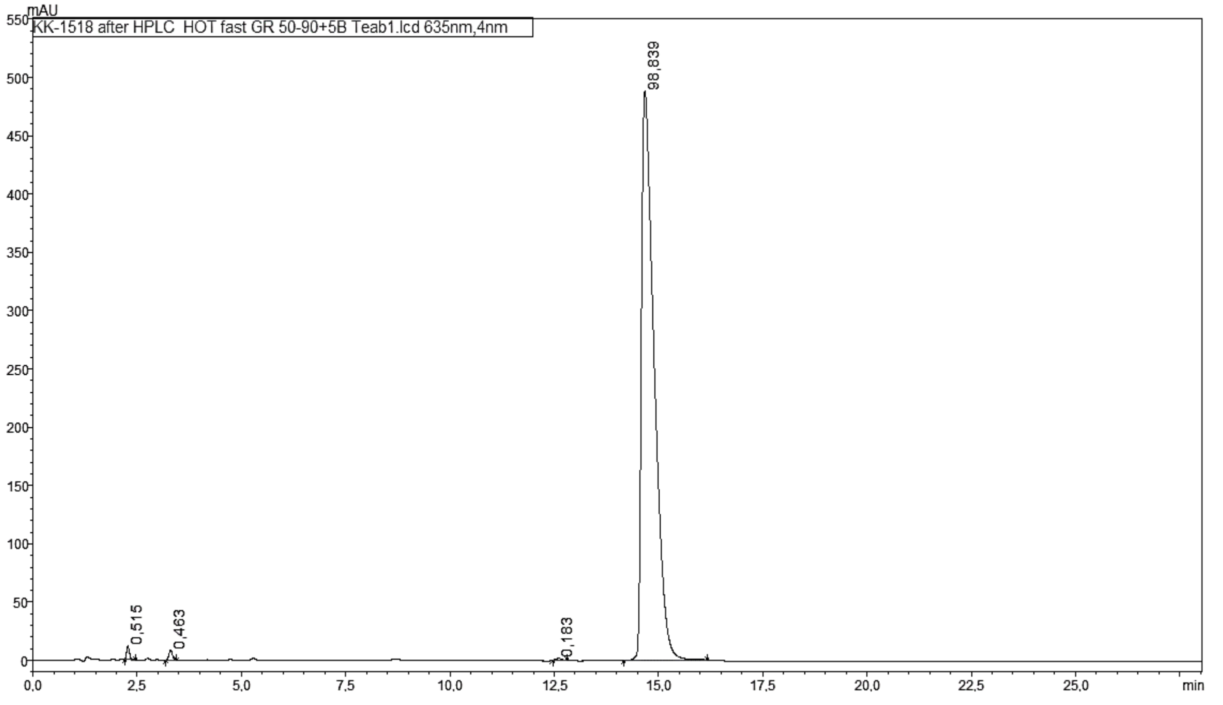

b

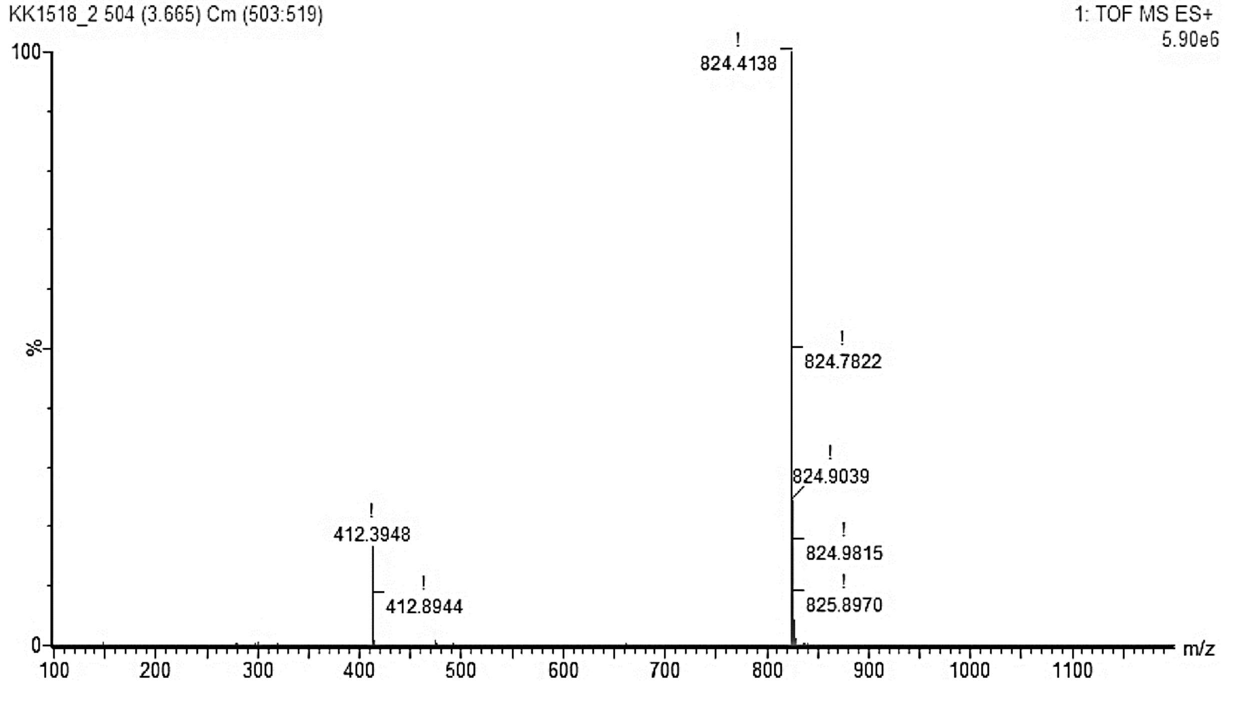

Fig. S8. HPLC analysis (**a**) and mass-spectrum (**b**) of the dye KK 1518.

a

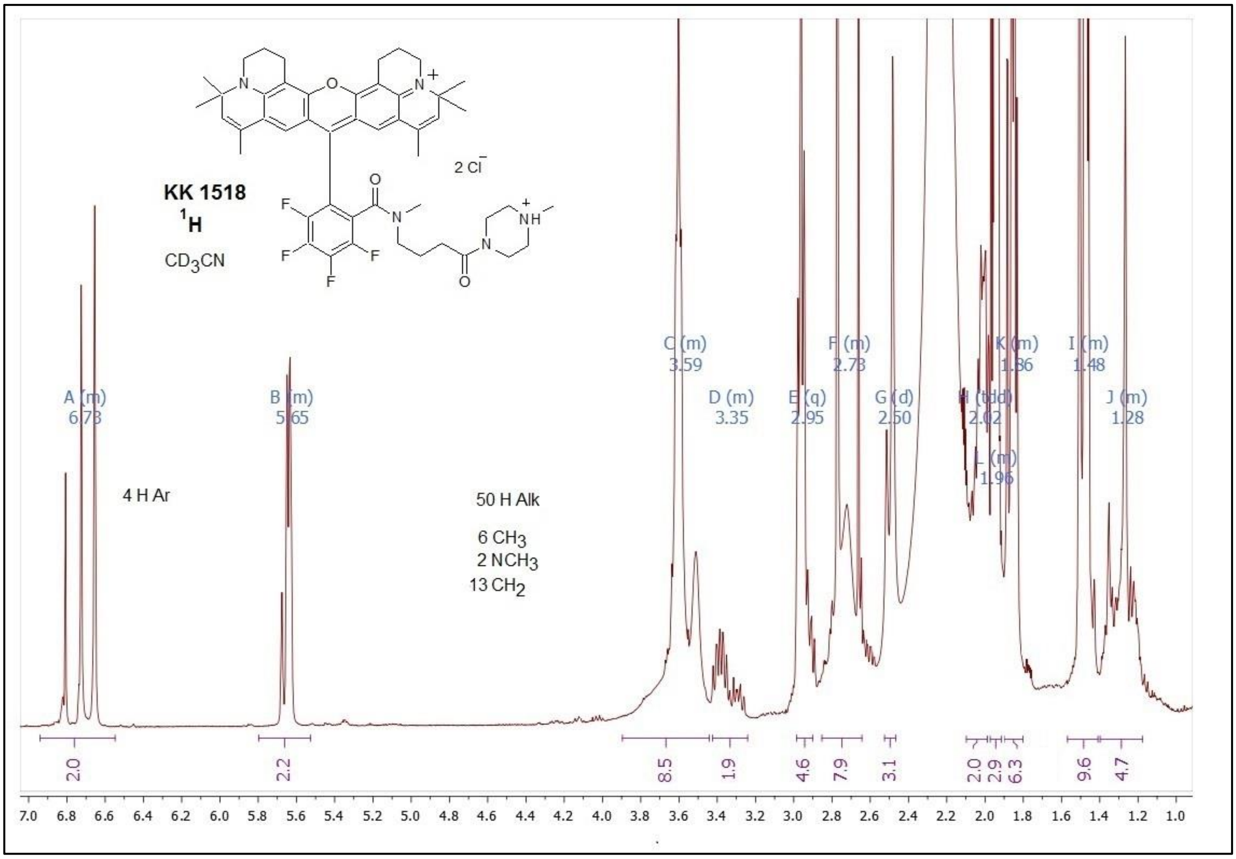
KK 1518
1H
CD3CN
2 Cl−
4 H Ar
50 H Alk
6 CH3
2 NCH3
13 CH2
A (m) 6.73
B (m) 5.65
C (m) 3.59
D (m) 3.35
E (q) 2.95
F (m) 2.73
G (d) 2.50
H (tdd) 2.02
L (m) 1.96
K (m) 1.86
I (m) 1.48
J (m) 1.28
2.0
2.2
8.5
1.9
4.6
7.9
3.1
2.0
2.9
6.3
9.6
4.7

b

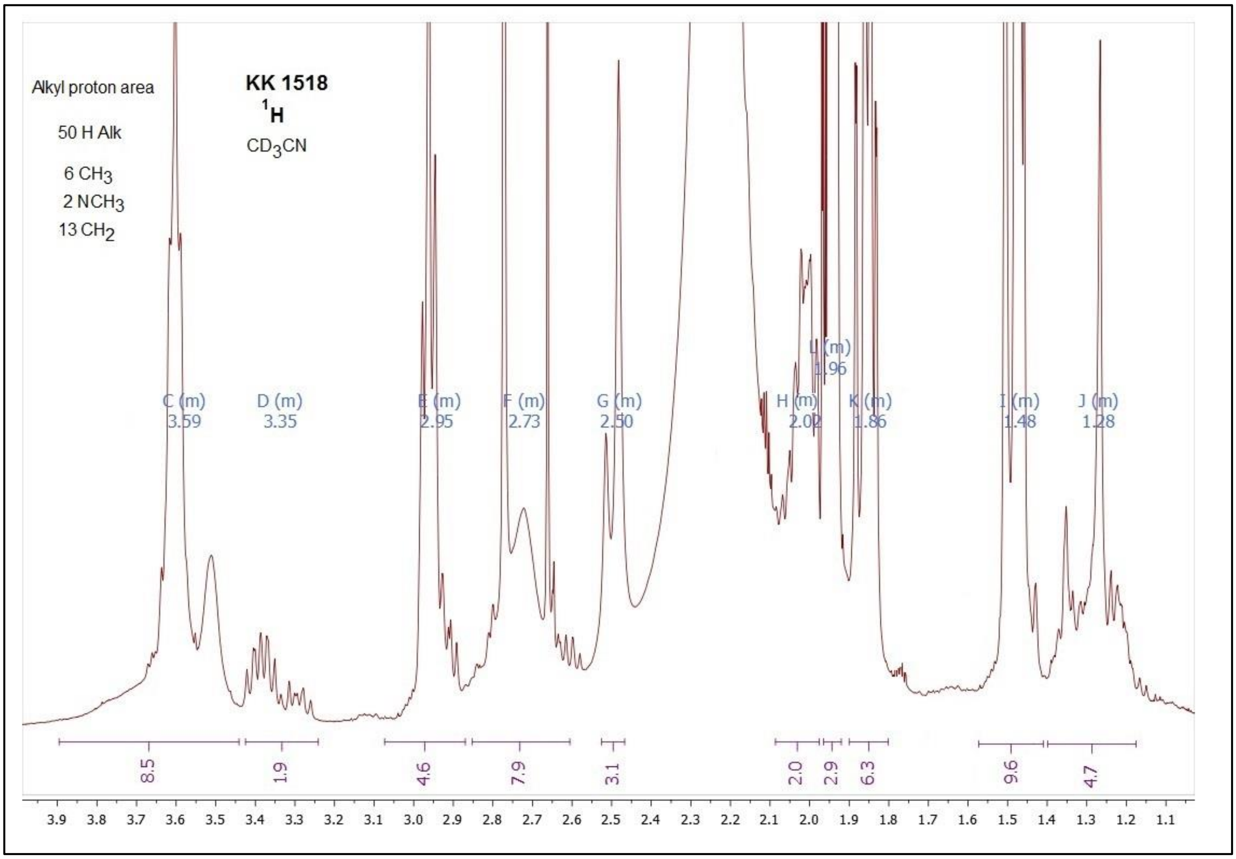
Alkyl proton area
50 H Alk
6 CH3
2 NCH3
13 CH2
KK 1518
1H
CD3CN
C (m) 3.59
D (m) 3.35
E (m) 2.95
F (m) 2.73
G (m) 2.50
H (m) 2.02
L (m) 1.96
K (m) 1.86
I (m) 1.48
J (m) 1.28
8.5
1.9
4.6
7.9
3.1
2.0
2.9
6.3
9.6
4.7

c

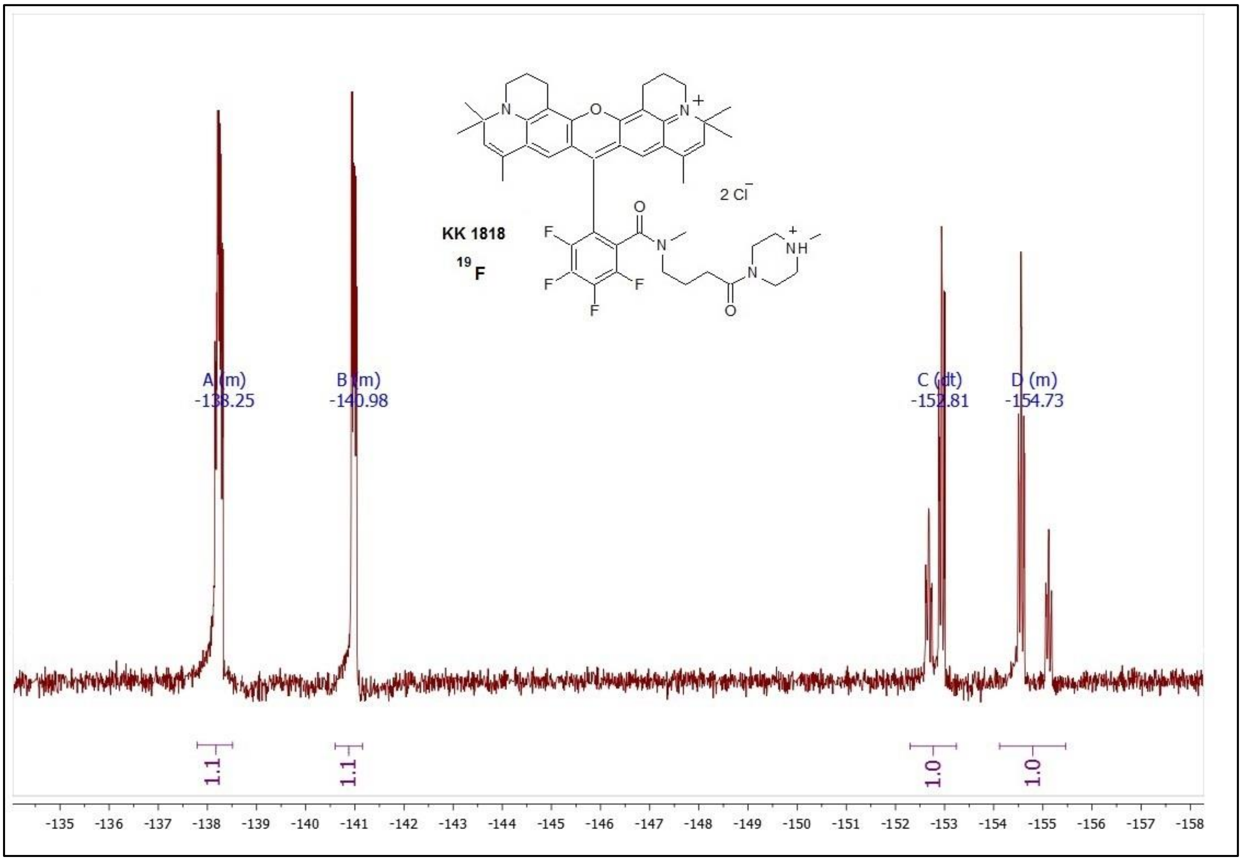

d

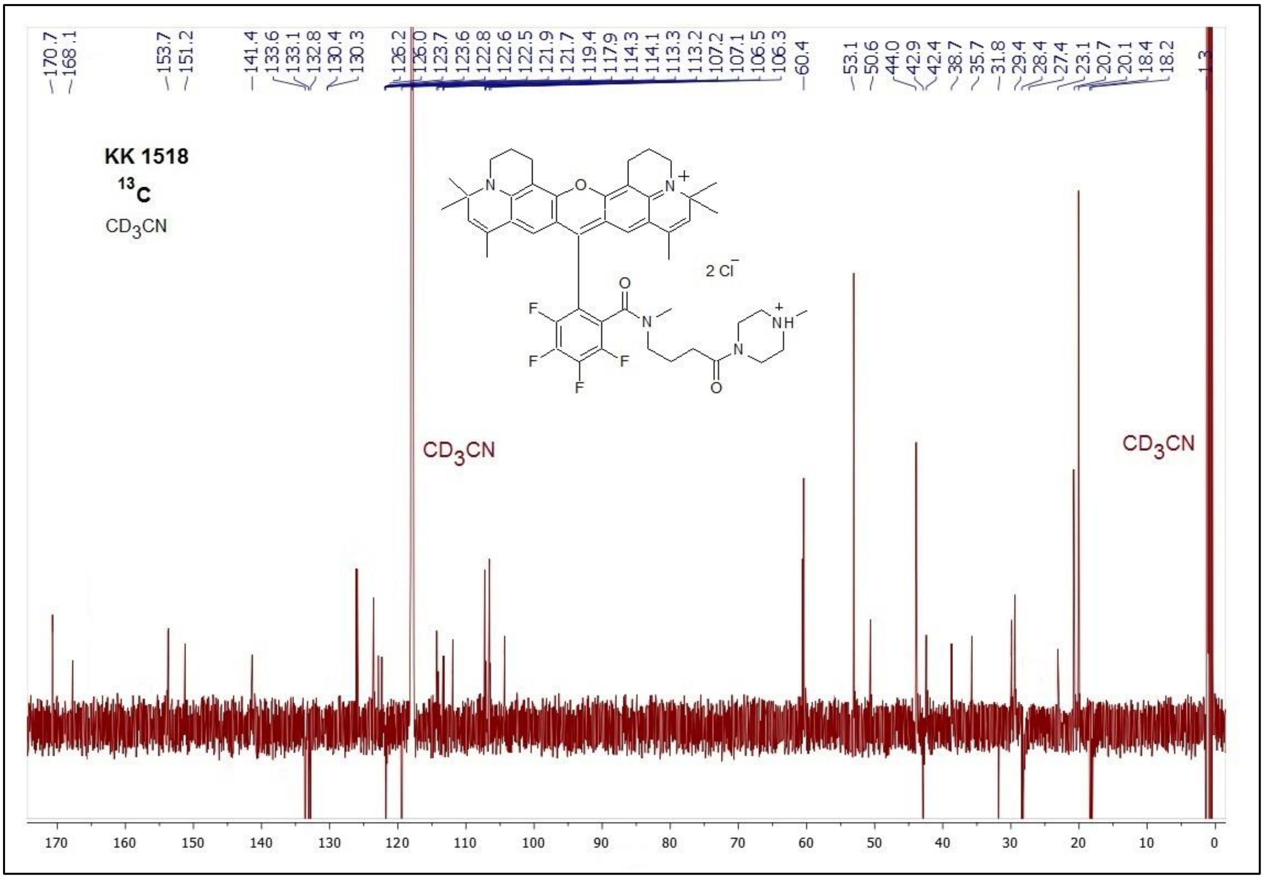

Fig. S9. NMR spectra of the dye KK 1518. Proton spectrum (**a**), with a focus on the alkyl proton area (**b**), fluorine spectrum (**c**) and carbon attached proton test (APT) spectrum (**d**) are shown.

a

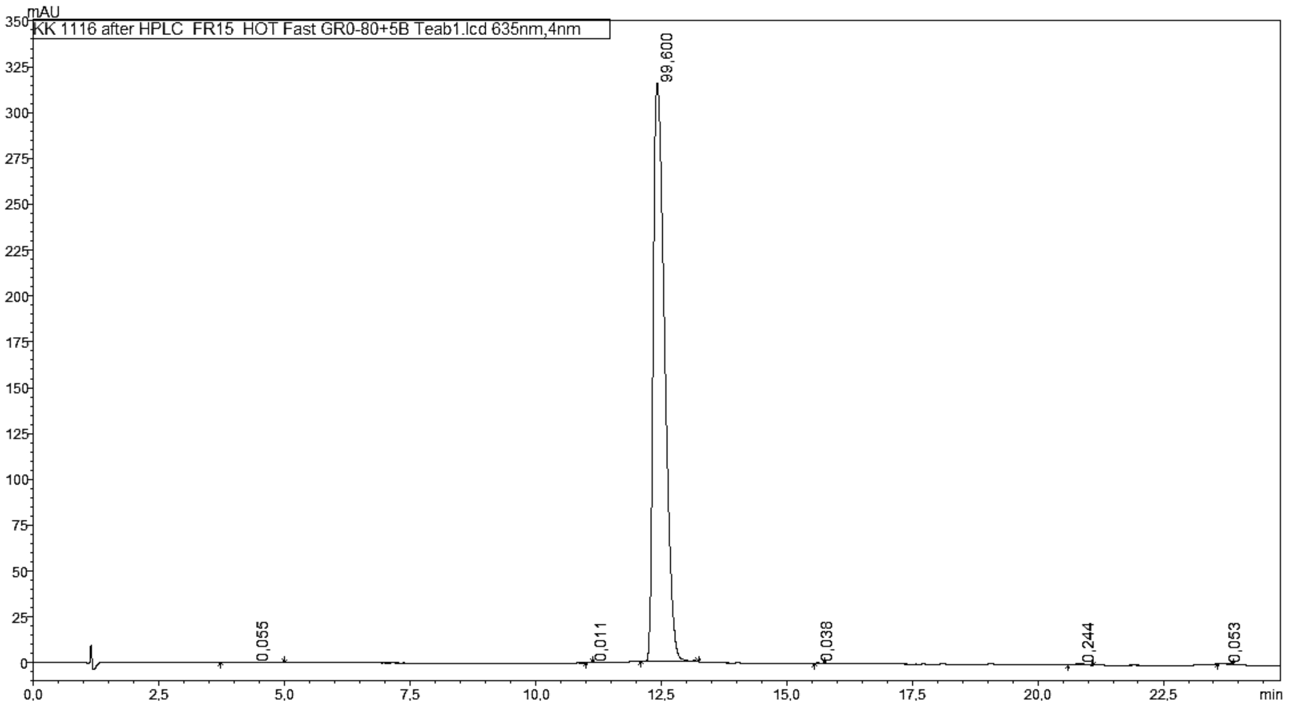

b

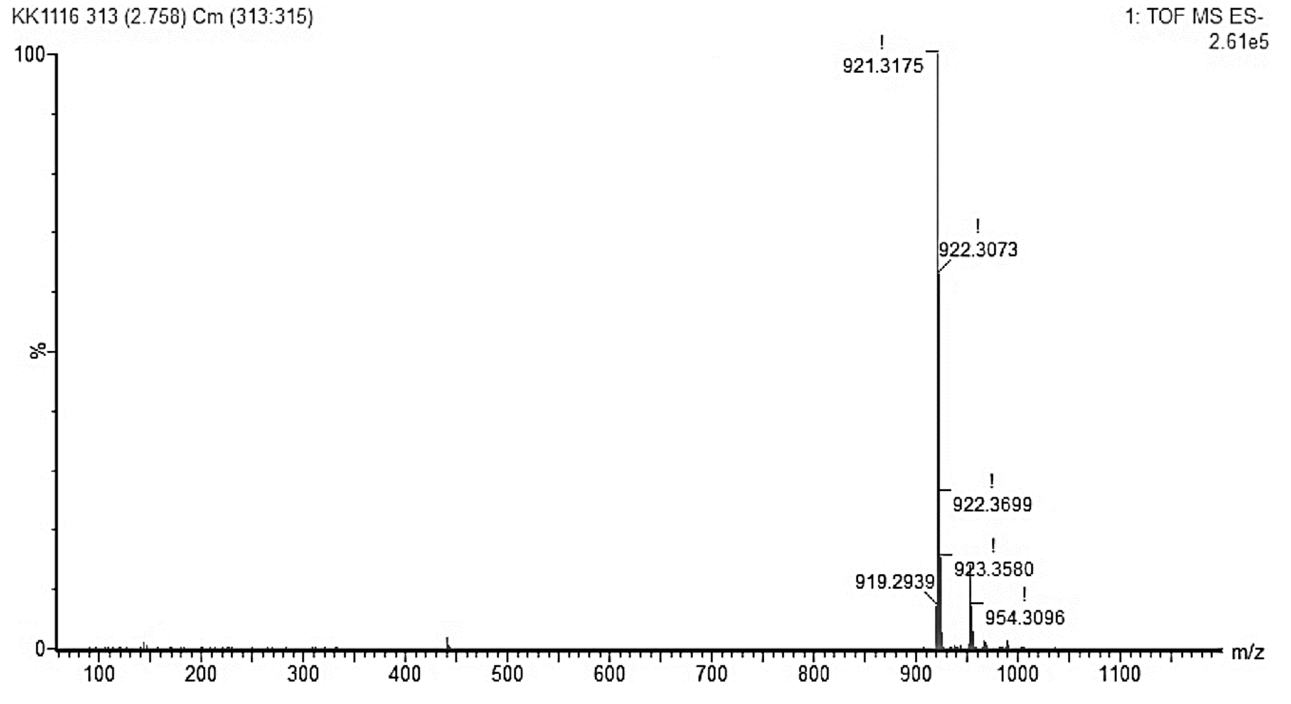

Fig. S10. HPLC analysis (**a**) and mass-spectrum (**b**) of the dye KK 1116.

a

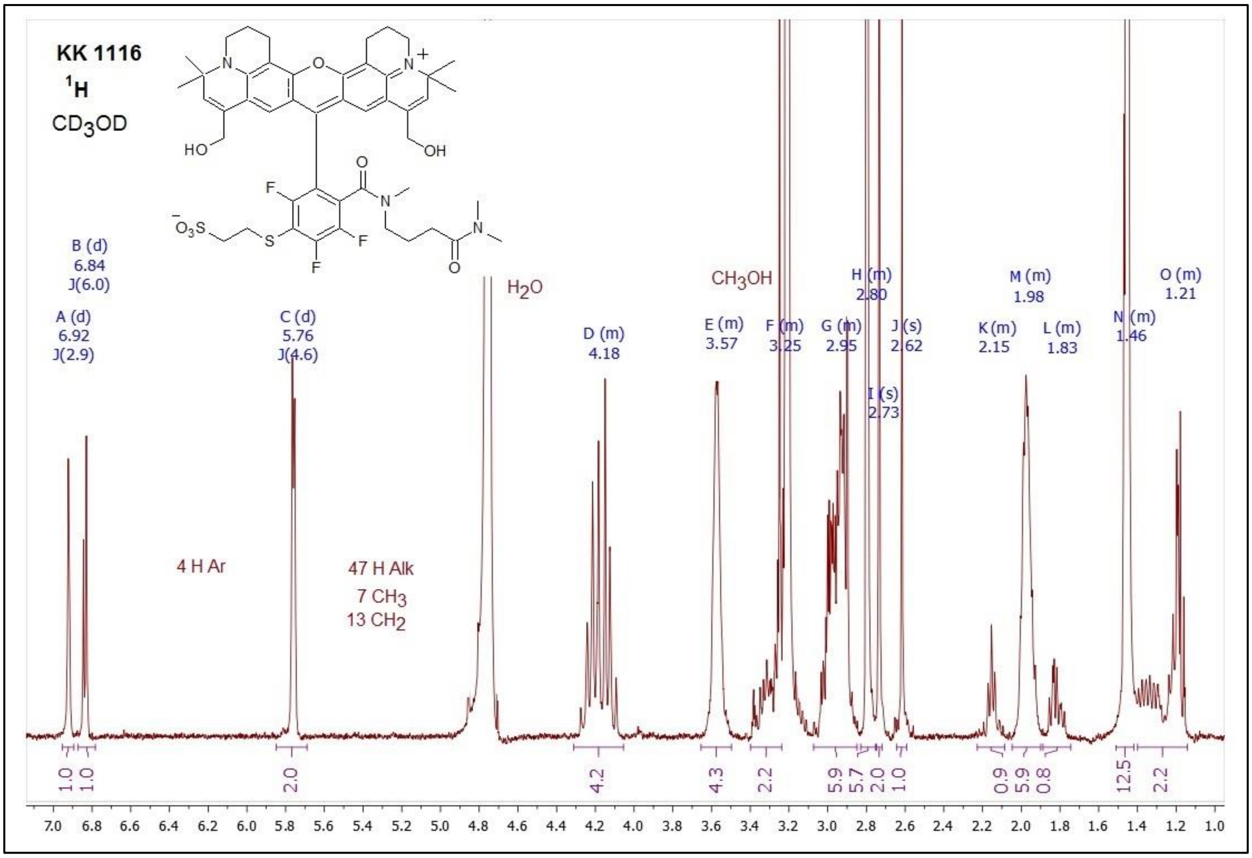

b

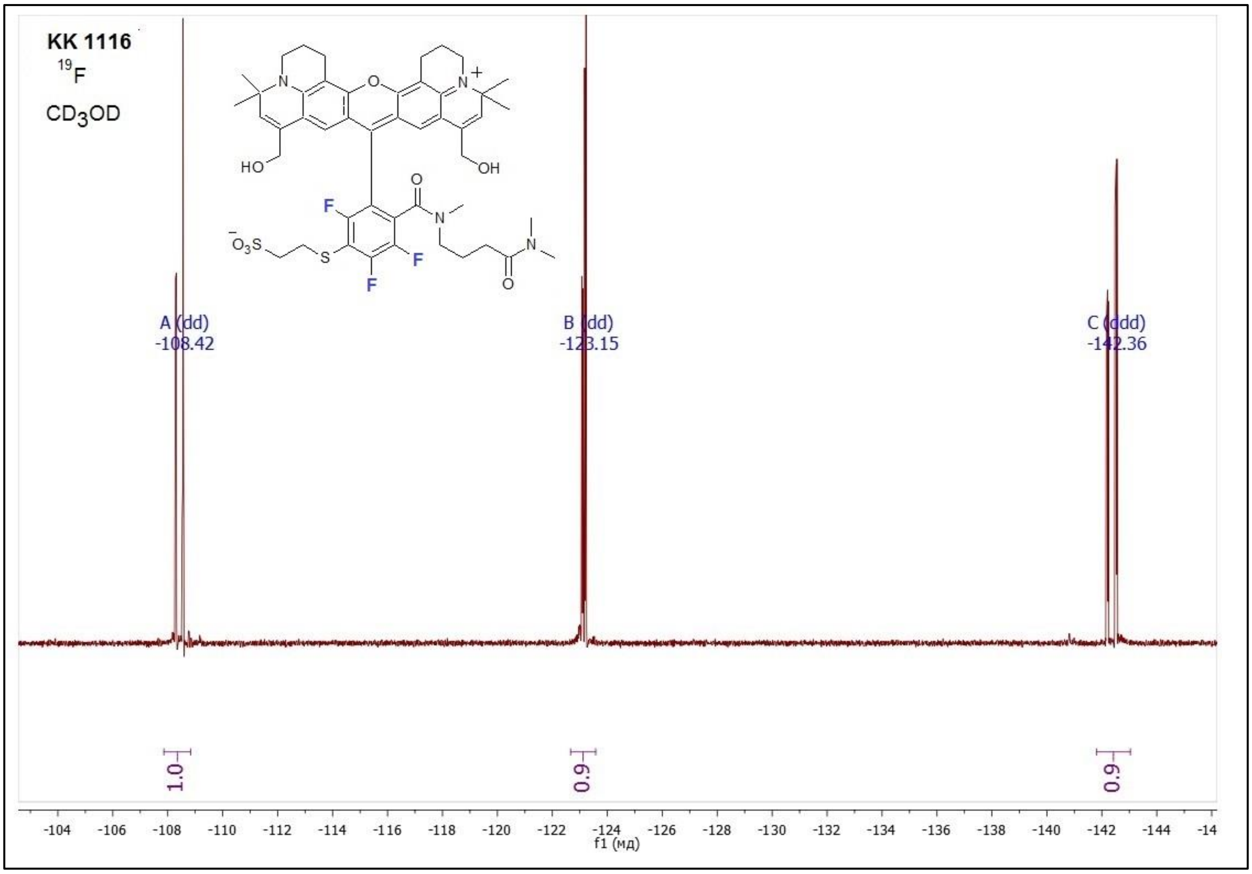

Fig. S11. NMR spectra of the dye KK 1116. Proton (**a**) and fluorine (**b**) spectra are shown.

a

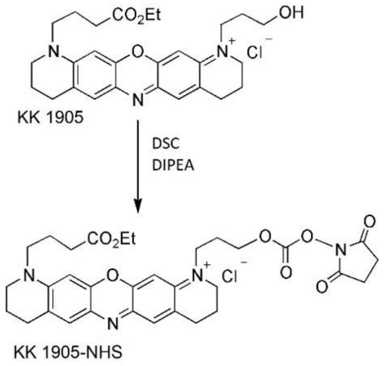

b

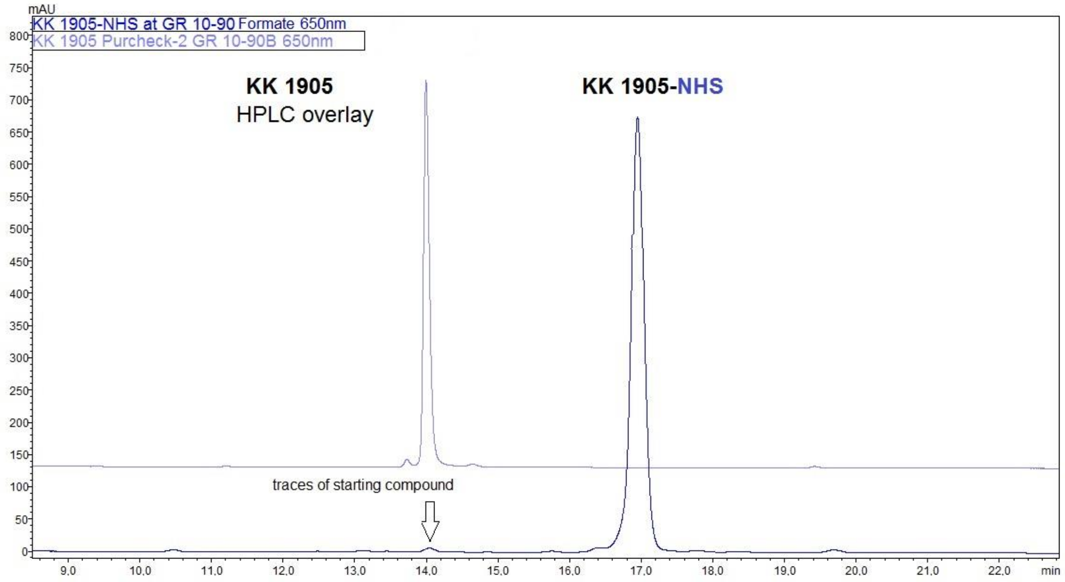

c

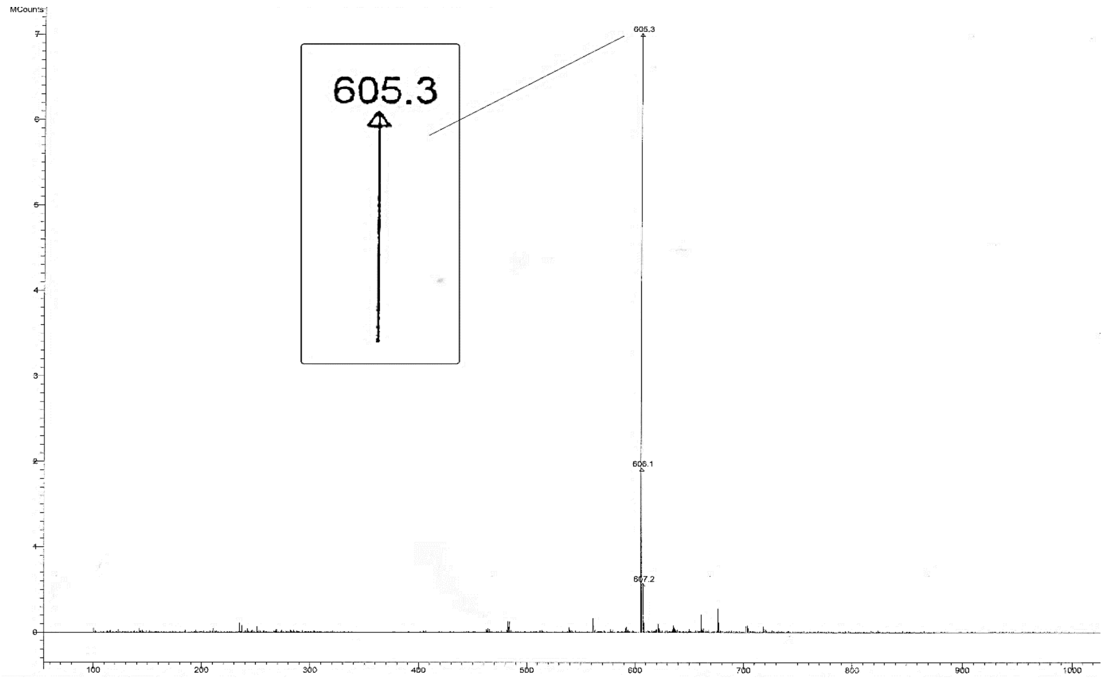

Fig. S12. (**a**) Reactions for the KK 1905-NHS generation from its precursor KK 1905. See Methods for other details. (**b**) HPLC analysis of the dye KK 1905 and its active NHS ester. (**c**) Mass-spectrum of the dye KK 1905-NHS.

a

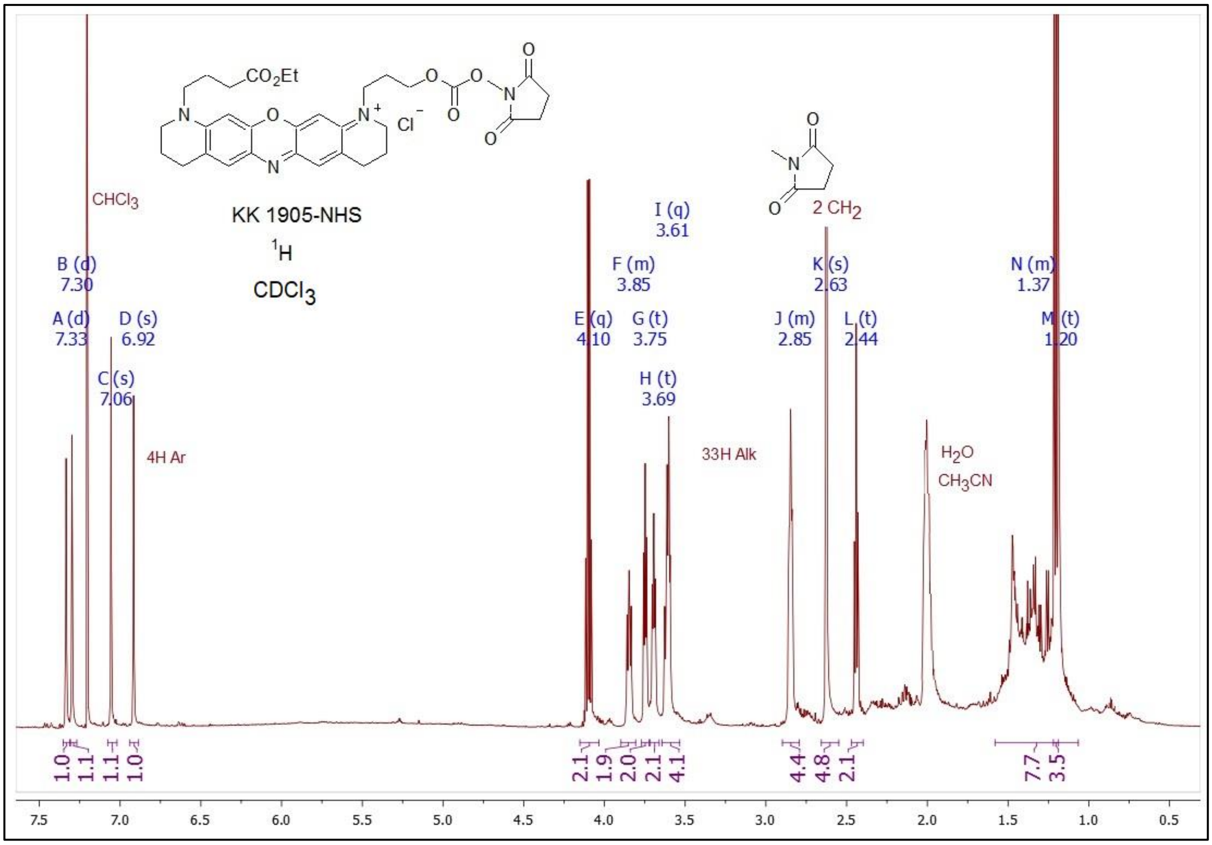
CO2Et
Cl⁻
KK 1905-NHS
1H
CDCl3
CHCl3
B (d)
7.30
A (d)
7.33
D (s)
6.92
C (s)
7.06
4H Ar
E (q)
4.10
F (m)
3.85
G (t)
3.75
H (t)
3.69
I (q)
3.61
33H Alk
J (m)
2.85
2 CH2
K (s)
2.63
L (t)
2.44
H2O
CH3CN
N (m)
1.37
M (t)
1.20
1.0 1.1 1.1 1.0 2.1 1.9 2.0 2.1 4.1 4.4 4.8 2.1 7.7 3.5
7.5 7.0 6.5 6.0 5.5 5.0 4.5 4.0 3.5 3.0 2.5 2.0 1.5 1.0 0.5

b

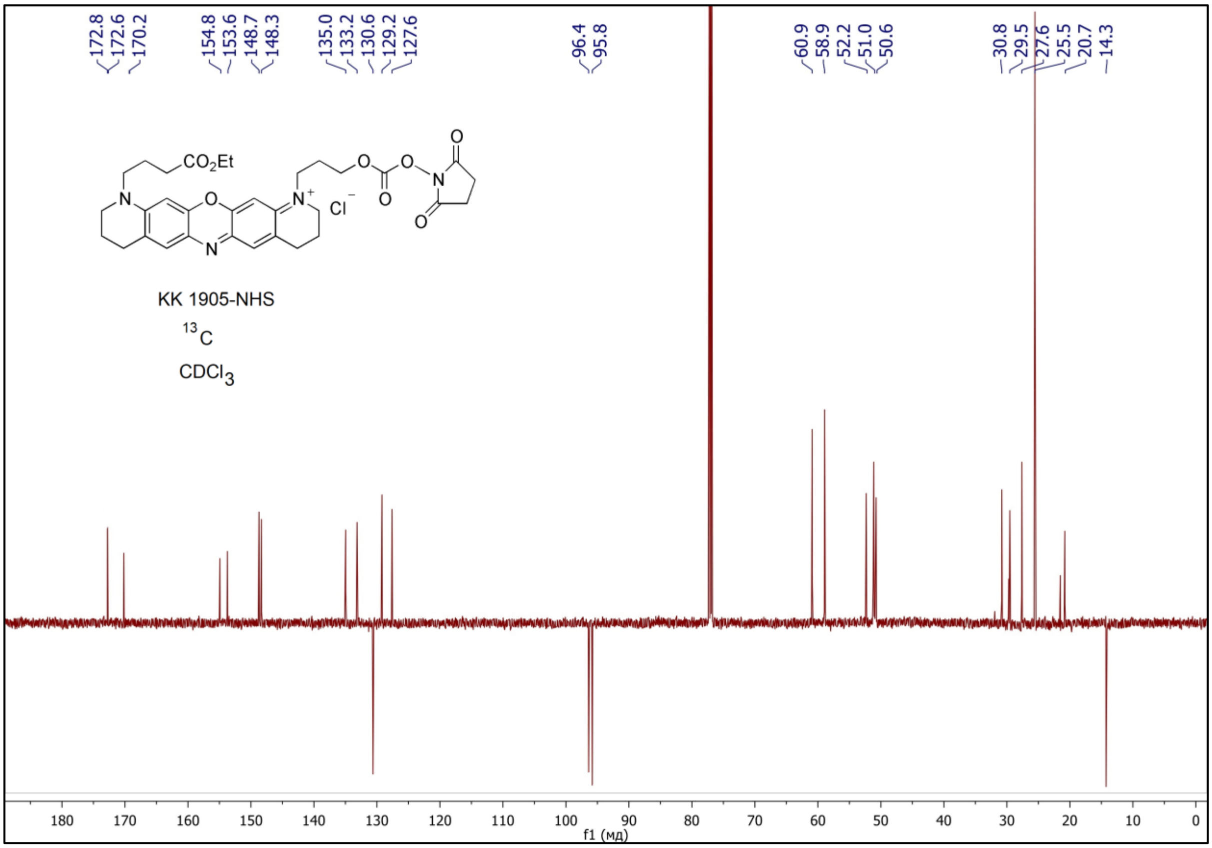
172.8
172.6
170.2
154.8
153.6
148.7
148.3
135.0
133.2
130.6
129.2
127.6
96.4
95.8
60.9
58.9
52.2
51.0
50.6
30.8
29.5
27.6
25.5
20.7
14.3
CO2Et
Cl⁻
KK 1905-NHS
13C
CDCl3
180 170 160 150 140 130 120 110 100 90 80 70 60 50 40 30 20 10 0
f1 (мд)

c

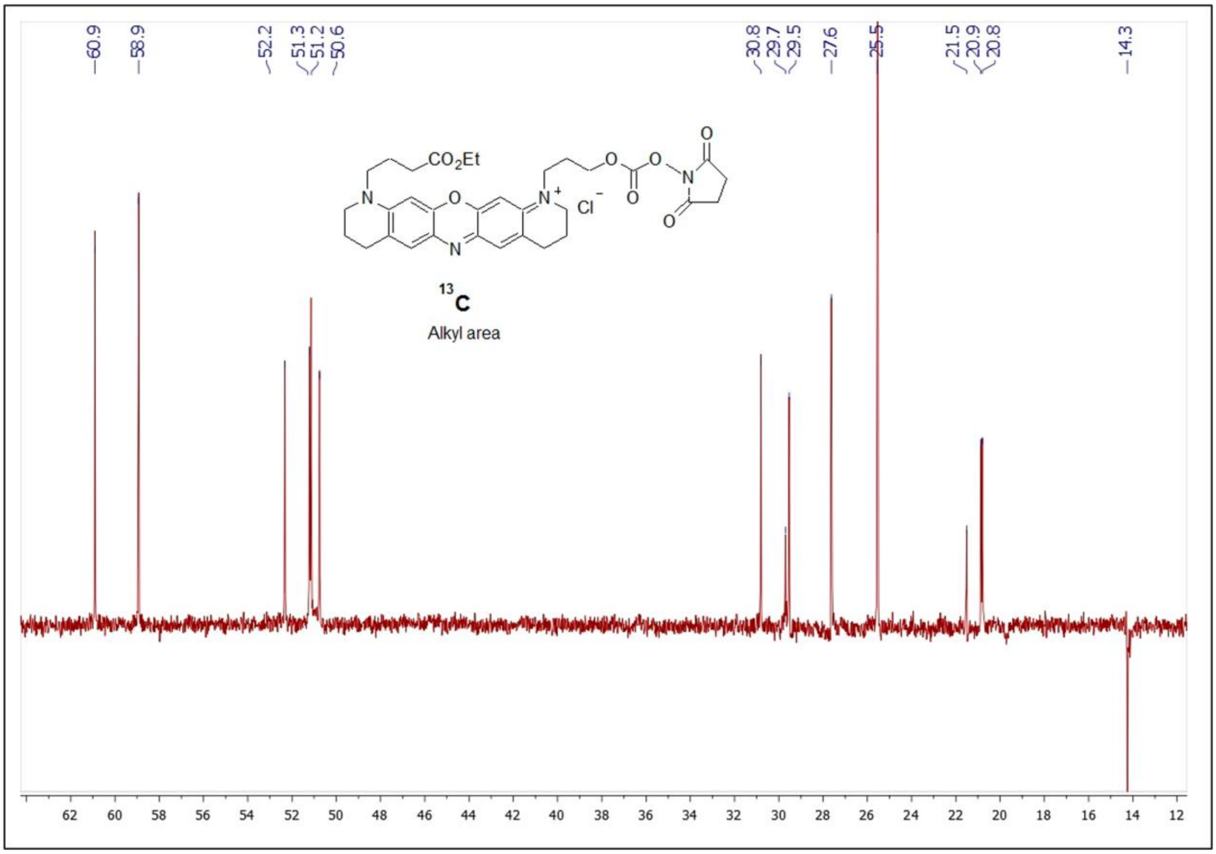

Fig. S13. NMR spectra of the dye KK 1905-NHS. Proton spectrum (**a**) and carbon APT spectrum (**b**), with a focus on the alkyl area (**c**) are shown.

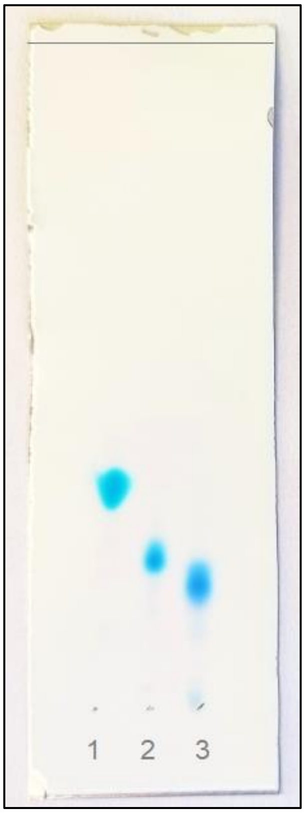

Fig. S14. TLC of three new compounds KK 1905-NHS (**1**), KK 1518 (**2**), and KK 1116 (**3**). Regular silica gel plates 60 F254 with a particle size of 10-12 mm were used with MeCN/DCM/H_2O, 10:1:1 + 0.2 vol. % TFA as mobile phase.

Table S1

Main fluorophores used for bacterial imaging

Fluorophore	λ_{abs} (nm)	λ_{em} (nm)	Application	Reference
DAPI	370	450	DNA staining	Hu *et al.*, 2017
JF 503	503	529	DNA staining	Foo *et al.*, 2015
JF 549	549	571	DNA staining	Foo *et al.*, 2015
JF 646	646	664	DNA staining	Foo *et al.*, 2015
SYTO 9	485	498	DNA staining	Berney *et al.*, 2007
Propidium iodide	535	617	DNA staining	Berney *et al.*, 2007
Ethidium bromide	301	603	DNA staining	Patel *et al.*, 2010
Hoechst	350	461	DNA staining	Simmons *et al.*, 2007
DMAO	496	528	DNA staining	Vasdekis *et al.*, 2013
Acridine orange [a]	500/460	526/650	DNA/RNA staining	Runci *et al.*, 2016
HADA	405	450	Peptidoglycan staining	Monteiro *et al.*, 2015
WGA-488	495	519	Peptidoglycan staining	Monteiro *et al.*, 2015
FM 1-43	470	579	Membrane staining	Bahlmann *et al.*, 2001
FM 4-64	515	728	Membrane staining	Spinnato *et al.*, 2022
KK114	638	655	Membrane staining	Lucidi *et al.*, 2020
Nile Red	554	638	Membrane and lipid droplets staining	Monteiro *et al.*, 2015
CTC	480	590	Reduced by the electron transport system; direct indicator of oxidative metabolism	Creach *et al.*, 2003

[a] Acridine orange is a cell-permeant nucleic acid binding dye that undergoes a bathochromic shift when bound to dsDNA (λ_{abs} = 500 nm; λ_{em} = 526 nm) and ssDNA or RNA (λ_{abs} = 460 nm; λ_{em} = 650 nm).

Table S2

CLSM settings employed for visualization of labeled bacterial samples

Dye	Sample treatment	PMT gain [a]	Power [b] (%)
KK 114S	Untreated	50	10
	Heat-inactivated	20	0.5
KK 114-NHS	Untreated	40	1
	Heat-inactivated	20	0.5
KK 1115	Untreated	50	5
	Heat-inactivated	30	1
KK 1116	Untreated	50	10
	Heat-inactivated	30	1
KK 1517	Untreated	40	1
	Heat-inactivated	30	0.5
KK 1517-NHS	Untreated	40	5
	Heat-inactivated	30	1
KK 1518	Untreated	50	2.5
	Heat-inactivated	30	1
KK 1558-NHS	Untreated	40	5
	Heat-inactivated	30	1
KK1905	Untreated	50	2.5
	Heat-inactivated	30	1
KK1905-NHS	Untreated	40	5
	Heat-inactivated	50	5
STAR RED	Untreated	45	5
	Heat-inactivated	30	1
PI	Untreated	50	5
	Heat-inactivated	30	1.5

[a] PMT gain and laser power were adjusted to maximize the signal/noise ratio. Pinhole size were maintained constant at 1.2 A. U.
[b] Laser power out of fibre.

Video captions

Video S1. Time-lapse of *B. subtilis* cells harvested from the death phase seeded on a LB 0.5% (w/v) agarose pad supplemented with KK 1905. The time each image was captured is shown at the bottom left. For each time point, DIC, KK 1905 fluorescence, and KK 1905 fluorescence-DIC merged channels are shown.

Video S2. Time-lapse of *B. subtilis* cells harvested from the death phase, stained with KK 1905-NHS, and seeded on a LB 0.5% (w/v) agarose pad. The time each image was captured is shown at the bottom left. For each time point, DIC, KK 1905-NHS fluorescence, and KK 1905-NHS fluorescence-DIC merged channels are shown.

General information for analytical data

ESI-MS spectra were obtained on a Varian 500 MS spectrometer (Agilent). High-resolution mass spectra (ESI-HRMS) were obtained on a Bruker micro TOF (ESI-TOF-MS) spectrometer. All masses are given in atomic units/elementary charge (*m/z*).

Nuclear Magnetic Resonance spectra were recorded using an Agilent 400-MR (^{1}H, ^{13}C) and a Bruker Avance Neo 600 spectrometer (^{19}F) in deuterated solvents at room temperature (298 K). Proton chemical shifts are reported in ppm (δ) relative to tetramethylsilane with the solvent resonance employed as the internal standard ($CDCl_3$, δ 7.26 ppm CD_3CN, δ 1.94 ppm). The following notation is used to describe the signal multiplicities: s = singlet, d = doublet, t = triplet, q = quartet, bs = broad singlet, dd = double doublet, ddd = double double doublet, dt = double triplet, ddt =double double triplet, dq = double quartet, td = triple doublet, and m = multiplet. Coupling constant (*J*) was expressed in Hz.

Shimadzu analytical HPLC system involved: an LC-20AD pump with a 4-channel low-pressure gradient valve unit 228-45040-58, degassing unit DGU-10A5R, and a diode-array detector SPD-M20A. Columns and phases are specified for each case. The preparative HPLC system consisted of an LC-20AP pump with a 4-channel low-pressure gradient valve unit FCV-10 AL, detector SPD-20AV, and a fraction collector FRC-10A. Chromatographic columns and phases are specified for each case. K. Kolmakov highly acknowledges BioBeagle Ltd and Peptide Technologies LLC (St. Petersburg, Russia) for the opportunity to prepare, analyze, and store the fluorophores.

Analytical data

KK 1518

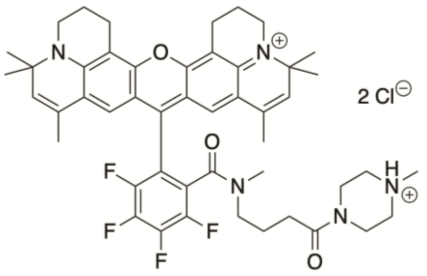

HPLC: t_R = 15 min (Kinetex C-18, 100 Å, 5 µm, 4.6 × 100 mm), 45 °C, gradient 50-90% B in 20 min, with 0.05 M aq. triethylammonium bicarbonate (TEAB) (A) and MeCN (B), flow rate 1.0 mL/min.

TLC: R_f = 0.20 (silica gel plates, MeCN/DCM/H_2O, 10:1:1 + 0.2 vol. % TFA).

LC/MS (ESI): *m*/*z* (positive mode, %) = 824 (90) $[M]^+$; high-resolution mass spectrometry (HRMS; $C_{48}H_{54}F_4N_5O_3$): 824.4138 (found M^+), 824.4163 (calc.).

^{1}H NMR (400 MHz, CD_3CN) * δ = 1.15 – 1.39 (m, CH_2, 1.28, s, NCH_3, 2 + 3H), 1.45 – 1.50 (m × 2, 9H, 3CH_3), 1.83 – 1.90 (m, 6H, 2CH_3), 1.96 (m, 3H, CH_3), 2.02 (m, 2H, CH_2), 2.66, 2.77 (s × 2, 3H, NCH_3), 2.68 – 2.85 (m, 4H, CH_2), 2.73 (m, 4H, 2NCH_2, piper.), 2.95 (m, 4H, CH_2), 3.35 (m, 2H, CH_2), 3.50 (m, 4H, 2NCH_2, piper.), 3.59 (m, 4H, NCH_2), 5.63 – 5.68 (m, 2H Ar), 6.65/6.73/6.82 (s × 3, 2H Ar) ppm.

^{19}F NMR (376.4 MHz, CD_3CN): δ = – 154.8 (m, 1 F), – 152.8 (dt, *J* = 100.1, 20.0 Hz, 1 F), – 141.0 (m, 1 F), – 138.2 (m, 1 F), ppm.

^{13}C NMR (100.6 MHz, CD_3CN): δ = 18.2 (CH_3), 18.4 (CH_3), 20.1 (CH_2), 20.7 (CH_2), 23.1 (CH_2), 27.4 (CH_3), 28.4 (CH_3), 29.4 (CH_2), 31.8 (CH_3), 35.7 (CH_2), 38.7 (CH_2), 42.4 (CH_2), 42.9 (CH_3), 44.0 (CH_2), 50.6 (CH_2), 53.1 (CH_2), 60.4 (CH_2), 106.3 (C), 106.5 (C), 107.1 (C), 107.2 (C), 113.2 (C), 113.3 (C), 114.1 (C), 114.3 (C), 119.4 (CH), 121.7 (C), 121.9 (CH), 122.5 (CH), 122.6 (C), 122.8 (C), 123.6 (C), 123.7 (C), 126.0 (C), 126.2 (C), 130.3 (CH), 130.4 (CH), 132.8 (CH), 133.1 (CH), 133.6 (CH), 141.4 (C), 151.2 (C), 151.2 (C), 153.7 (C), 168.1 (C=O), 170.7 (C=O) ppm.

* This compound is a mixture of amide rotamers with diastereotopic groups, which makes its NMR spectra complicated. Particularly, NCH_3 protons produce separate singlets (*e.g.*, dimethylformamide). The NMR spectra are well consistent with previously published data for the precursor compound KK 1517 [9].

Analytical data

KK 1116

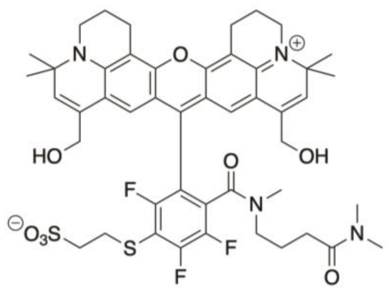

<u>HPLC</u>: t_R = 12.5 min (Kinetex C-18, 100 Å, 5 µm, 4.6 × 100 mm), 45 °C, gradient 0 – 80% B in 20 min, with 0.05 M aq. TEAB (A) and MeCN (B), flow rate 1.0 mL/min.

<u>TLC</u>: R_f = 0.17 (silica gel 60 F254 0.04-0.63 mm, MeCN/DCM/H_2O, 10:1:1 + 0.2 vol. % TFA).

<u>LC/MS (ESI)</u>: *m*/*z* (negative mode, %) = 921 (90) [M-H]; HRMS ($C_{47}H_{52}F_3N_4O_8S_2$): 921.3175 (found M-H), 921.3179 (calc.).

<u>^{1}H NMR</u> (400 MHz, CD_3OD) *: δ = 1.15 – 1.40, (m, 2H, CH_2), 1.46 (m, 12H, 4CH_3), 1.98 (m, 6H, 3CH_2), 1.83, 2.15 (m, × 2, 2H, CH_2), 2.62, 2.73 (s × 2, 3H, NCH_3), 2.79, 2.80 (s × 2, 6H, $N(CH_3)_2$), 2.90 – 3.05 (m, 6H, 3CH_2), 3.23 – 3.40 (br. m, 2H, CH_2, CH_2SO_3), 3.57 (m, 4H, 2CH_2), 4.10 – 4.30 (m, 4H, 2CH_2OH), 5.76 (d, *J* = 6 Hz, 2H), 6.84 (d, *J* = 6 Hz, 1H), 6.92 (d, *J* = 2.9 Hz, 1H) ppm;

<u>^{19}F NMR</u> (376.4 MHz, CD_3OD): δ = – 108.42 (dd, 1 F, *J* = 142.1, 13.7 Hz), – 123.15 (dd, 1 F, *J* = 58.9, 24.7 Hz , – 142.36 (ddd, 1 F, *J* = 178.2, 24.7, 14.0 Hz) ppm.

* This compound is a mixture of amide rotamers with diastereotopic groups, which makes NMR spectra complicated. Particularly, NCH_3 protons produce separate singlets (*e.g.*, dimethylformamide). The NMR spectra are well consistent with previously published data for a similar compound named KK 1103 (see supplementary material from ref. [9]) and with those for its precursors (see compounds 4a and 6-H in the supplementary material of Kolmakov *et al.*, 2012)

Analytical data

KK 1905-NHS

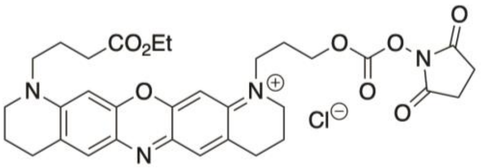

HPLC: t_R = 19.5 min (Kinetex C-18, 100 Å, 5 µm, 4.6 × 100 mm), 25 °C, gradient 10 – 90% B in 20 min, with a triethylammonium formate buffer with pH = 3.2 containing 0.01 M triethylammonium formate and 0.04 M formic acid (A) and MeCN (B), flow rate 0.5 mL/min.

TLC: R_f = 0.25 (silica gel plates, DCM/MeOH, 8:1) or 0.40 (with MeCN/DCM/H_2O, 10:1:1 + 0.2 vol. % TFA).

LC/MS (ESI): *m*/*z* (positive mode, %) = 605 (80) $[M]^+$; HRMS ($C_{32}H_{37}N_4O_8$): 605.2623 (found M+), 605.2611 (calc.).

^{1}H NMR (400 MHz, $CDCl_3$): δ = 1.20 (t, *J* = 7 Hz, 3H, $CH_2\underline{CH_3}$), 1.06 – 1.58 (m, 8H, 4CH_2), 2.44 (t, *J* = 7 Hz, 4H, CH_2), 2.63 (s, 4H, 2CH_2CO), 2.85 (m, 4H, 2CH_2), 3.69 (t, *J* = 5.8 Hz, 2H), 3.75 (t, *J* = 6 Hz, 2H), 3.80 – 3.90 (m, 2H), 4.10 (q, *J* = 7 Hz, 2H, OEt), 6.92 (s, 1H), 7.06 (s, 1H), 7.30 (d, *J* = 1.5 Hz, 1H), 7.33 (d, *J* = 1.5 Hz, 1H) ppm.

^{13}C NMR (100.6 MHz, $CDCl_3$): δ = 14.3 (CH_3), 20.7 (CH_2), 21.5 (CH_2), 25.5 (CH_2), 27.6 (CH_2), 29.5 (CH_2), 29.7 (CH_2), 30.8 (CH_2), 50.6 (CH_2), 51.2 (CH_2), 51.3 (CH_2), 52.3 (CH_2) 58.9 ($\underline{CH_2}$O), 60.9 ($\underline{CH_2}$CO), 95.8 (CH), 96.4 (CH), 127.6 (C), 129.2 (C), 130.6 (CH), 133.2 (C), 135.0 (C), 148.3 (C), 148.7 (C) 153.6 (C), 154.8 (C), 170.2, (C=O), 172.6, (C=O), 172.8 (C=O) ppm.

The spectra are well consistent with previously published data for the precursor compound KK 1905 (see Compound 20-Et-H in the supplementary material of ref. [8]).

Supplementary references

Bahlmann K, Jakobs S, Hell SW. 2001. 4Pi-confocal microscopy of live cells. Ultramicroscopy 87:155–164.

Berney M, Hammes F, Bosshard F, Weilenmann H-U, Egli T. 2007. Assessment and interpretation of bacterial viability by using the LIVE/DEAD BacLight Kit in combination with flow cytometry. Appl Environ Microbiol 73:3283–3290.

Créach V, Baudoux AC, Bertru G, Rouzic BL. 2003. Direct estimate of active bacteria: CTC use and limitations. J Microbiol Methods 52:19–28.

Foo YH, Spahn C, Zhang H, Heilemann M, Kenney LJ. 2015. Single cell super-resolution imaging of *E. coli* OmpR during environmental stress. Integrative Biology 7:1297–1308.

Hu W, Murata K, Zhang D. 2017. Applicability of LIVE/DEAD BacLight stain with glutaraldehyde fixation for the measurement of bacterial abundance and viability in rainwater. J Environ Sci (China) 51:202–213.

Kolmakov K, Wurm CA, Hennig R, Rapp E, Jakobs S, Belov VN, Hell SW. 2012. Red-emitting rhodamines with hydroxylated, sulfonated, and phosphorylated dye residues and their use in fluorescence nanoscopy. Chemistry 18:12986–12998.

Lucidi M, Hristu R, Nichele L, Stanciu GA, Tranca DE, Holban AM, Visca P, Stanciu SG, Cincotti G. 2020. STED nanoscopy of KK114-stained pathogenic bacteria. J Biophotonics 13:e202000097.

Monteiro JM, Fernandes PB, Vaz F, Pereira AR, Tavares AC, Ferreira MT, Pereira PM, Veiga H, Kuru E, VanNieuwenhze MS, Brun YV, Filipe SR, Pinho MG. 2015. Cell shape dynamics during the staphylococcal cell cycle. Nat Commun 6:8055.

Patel D, Kosmidis C, Seo SM, Kaatz GW. 2010. Ethidium bromide MIC screening for enhanced efflux pump gene expression or efflux activity in *Staphylococcus aureus*. Antimicrob Agents Chemother 54:5070–5073.

Runci F, Bonchi C, Frangipani E, Visaggio D, Visca P. 2016. *Acinetobacter baumannii* biofilm formation in human serum and disruption by gallium. Antimicrob Agents Chemother 61:e01563-16.

Simmons W, Bolland J, Daubenspeck J, Dybvig K. 2007. A stochastic mechanism for biofilm formation by *Mycoplasma pulmonis*. Journal of Bacteriology 189:1905–13.

Spinnato MC, Lo Sciuto A, Mercolino J, Lucidi M, Leoni L, Rampioni G, Visca P, Imperi F. 2022. Effect of a defective clamp loader complex of DNA polymerase III on growth and SOS response in *Pseudomonas aeruginosa*. Microorganisms 10:423.

Vasdekis AE. 2013. Single microbe trap and release in sub-microfluidics. RSC Adv 3:6343–6346.
